# A randomized controlled trial of a 14-day mindfulness ecological momentary intervention (MEMI) for generalized anxiety disorder

**DOI:** 10.1192/j.eurpsy.2023.2

**Published:** 2023-01-16

**Authors:** Nur Hani Zainal, Michelle G. Newman

**Affiliations:** 1 Department of Health Care Policy, Harvard Medical School, Boston, Massachusetts, USA; 2 Department of Psychology, The Pennsylvania State University, State College, Pennsylvania, USA; 3 Department of Psychology, National University of Singapore, Singapore

**Keywords:** ecological momentary intervention, executive function, generalized anxiety disorder, mindfulness, randomized controlled trial

## Abstract

**Background:**

Little is known about whether brief mindfulness ecological momentary interventions (MEMIs) yield clinically beneficial effects. This gap exists despite the rapid growth of smartphone mindfulness applications. Specifically, no prior brief MEMI has targeted generalized anxiety disorder (GAD). Moreover, although theories propose that MEMIs can boost executive functioning (EF), they have largely gone untested. Thus, this randomized controlled trial (RCT) aimed to address these gaps by assessing the efficacy of a 14-day smartphone MEMI (versus self-monitoring placebo [SMP]).

**Method:**

Participants with GAD were randomly assigned to either condition (68 MEMI and 42 SMP). MEMI participants exercised multiple core mindfulness strategies and were instructed to practice mindfulness continually. Comparatively, SMP participants were prompted to practice self-monitoring and were not taught any mindfulness strategies. All prompts occurred five times a day for 14 consecutive days. Participants completed self-reports and neuropsychological assessments at baseline, posttreatment, and 1-month follow-up (1MFU). Piecewise hierarchical linear modeling analyses were conducted.

**Results:**

MEMI (versus SMP) produced greater pre-1MFU reductions in GAD severity and perseverative cognitions (between-group *d* = 0.393–0.394) and stronger improvements in trait mindfulness and performance-based inhibition (*d* = 0.280–0.303). Further, MEMI (versus SMP) led to more considerable pre- to posttreatment reduction in state-level depression and anxiety and more mindfulness gains (*d* = 0.50–1.13). Overall, between-treatment effects were stronger at pre-1MFU than pre- to posttreatment for trait-level than state-level treatment outcome measures.

**Conclusions:**

Preliminary findings suggest that the beneficial effect of an unguided brief MEMI to target pathological worry, trait mindfulness, and EF is modest yet potentially meaningful. Other theoretical and clinical implications were discussed.

## Introduction

Frequently, people engage in activities on autopilot [1] or avoid discomfort [2]. Such mindless states that prevail in our daily lives could create negative consequences. For instance, a study found that our minds wandered 47% of waking hours, and mind-wandering predicted future unhappiness [3]. Comparatively, being mindful (i.e., receptively, flexibly, and nonjudgmentally focusing on the present continually) [4] was related to higher daily positive affect and well-being [5]. Mindfulness was also associated with experiential, behavioral, and neural emotion regulation processes [6]. Thus, it has important implications for the cognitive and clinical sciences.

Practicing mindfulness could improve well-being and alleviate common mental health symptoms via diverse pathways. Mindfulness theories posit that persistently exercising present-moment awareness, acceptance, and related skills reduces suboptimal stress reactivity [7, 8]. Specific mindfulness practices (e.g., continually engaging in values-consistent activities) may confer salutary psychological effects by enhancing self-compassion and self-efficacy [9, 10]. Over time, mindfulness can help people experience declines in depression and anxiety symptoms via a more adaptive way of relating to the ebb and flow of their cognitions, feelings, and physical sensations [11]. Identifying and disengaging from unhelpful thoughts and actions by using alternative mindfulness skills are integral to the process [12].

Further, the ongoing practice of mindfulness relates closely to higher-order cognitive processes. As being mindful makes one more cognizant and receptive of all elements in the experiential field, mindfulness-based interventions (MBIs) are theorized to enhance attention and executive functioning (EF; e.g., *monitoring and acceptance theory*) [13, 14]. EF refers to the multi-domain, higher-order, cognitive control capacity to strategically initiate, plan, and maintain goal-directed actions and adapt swiftly to unanticipated events [15]. Thus, EF is essential for optimally regulating various cognitive and behavioral processes. One EF skill includes *inhibition* (capacity to refrain from autopilot responding) [16]. MBIs teach individuals to deliberately sustain attention to a unique aspect of experience (e.g., task-at-hand), refocus following any distractions, and repeatedly notice and act the opposite of unproductive urges. Thus, MBIs are thought to enhance attention regulation and EF and improve inhibition [17].

One way to advance clinical science is to test the efficacy of mindfulness ecological momentary interventions (MEMIs) on EF and symptoms in psychiatric samples. MEMIs can repeatedly instruct patients to inhibit judgment and other unhelpful impulses or habits and harness EF to deploy mindfulness strategies in *real time* in various situations. Patients may thus experience symptom changes by being more mindful and adaptable [18, 19]. Further, most people with mental health problems own a smartphone and are receptive to mobile health therapies [20]. For instance, as generalized anxiety disorder (GAD) sufferers reported shame, monetary, organizational, and logistical concerns about seeking face-to-face treatment [21], unguided EMIs might allow privacy, portability, and flexibility to manage worries and related symptoms independently. Also, as MEMIs have been proliferating in recent years (e.g., iMindfulness) [22], it is important to examine their efficacy. Few EMIs have been studied in randomized controlled trials (RCTs) [23]. Also, MEMIs may close current treatment gaps [24]. For these reasons, researching, refining, and disseminating empirically supported MEMIs are essential.

To fine-tune MEMIs, a fruitful endeavor may be to investigate their potential to produce therapeutic benefits across *brief* durations, defined as a single session or repeated exposure to the intervention for up to 2 weeks [25]. An RCT by LaFreniere and Newman [49] showed that 10 days of tracking worries, chances of anticipated fears occurring, and actual outcomes (versus recording thoughts) four times a day reduced trait worry in GAD. Treatment gains were maintained at the 30-day follow-up. It also showed notable between-group differences in the proportion of participants meeting GAD diagnostic criteria posttreatment but not at follow-up. Relatedly, a 7-day audio-based self-guided MBI plus working memory (WM) training (versus control) reduced worry and increased WM in high worriers [26]. These brief treatments reflect the exception rather than the norm. Most EMIs conducted so far targeted depression [27] and involved long therapy sessions, therapist contact, and standard cognitive-behavioral therapy (CBT) [23, 28–30]. Little is known about short-term, low-intensity, self-help MEMIs. Standard 1–2.5 h weekly across 8–16 session MBIs require sizeable monetary and time commitment [31]. Thus, brief MEMIs can defray such costs and help people effectively regulate acute stress in various settings [32].

Despite the potential advantages of brief MEMIs, only five RCTs have examined their impact. First, a 10-day MEMI that instructed mindfulness exercises three times per day enhanced daily mindfulness, sleep length, and quality in a convenience sample of stressed workers [33]. However, as it did not include an active control group, threats to internal validity (e.g., regression to the mean, expectancy effects) could not be ruled out. Second, a 14-day MEMI (versus thought wandering control) reduced nicotine craving and consumption in heavy smokers [34]; however, between-group effects on negative affect (NA) may not have been found due to the small sample size. Another three RCTs tested the efficacy of Headspace, an app-delivered series of mindfulness techniques used for 10 min each day across 10 days. Also, the Headspace app (versus list-making) led to changes in depression and positive affect in middle-aged, happiness-seeking adults; nonetheless, expected changes in NA, life satisfaction, and flourishing were not found [35]. Additionally, two widely used MEMIs (Headspace and Smiling Mind versus activity listing) reduced depressive symptoms and enhanced college adjustment (but not anxiety, stress, and flourishing) at posttreatment and 30-day follow-up among undergraduates [36]. Further, Headspace (versus waitlist) raised life satisfaction and resilience and decreased stress in workplace employees [37].

These prior MEMI RCTs also have three other shortcomings. First, none recruited persons with any mental health disorder clinical symptoms. Thus, there is a need for more studies to determine if such results can be extrapolated to clinical samples. Second, the studies testing popular apps, instructed mindfulness practices for only one, 10- or 20-min/day. It is, therefore, unclear if findings would be replicated or improved upon in a MEMI that prompted clinically distressed participants *multiple* times daily. Third, these apps still incur considerable customer costs (e.g., $95.88–$239.88 for an annual subscription to Headspace). Thus, a nominally costing MEMI with the same benefits as these subscription apps would be valuable.

Accordingly, this study tested the efficacy of a 14-day MEMI package compared with a self-monitoring placebo (SMP) for persons with GAD. Maintenance of gains was assessed at a 1-month follow-up (1MFU). We hypothesized that MEMI (versus SMP) would significantly reduce GAD severity and perseverative cognitions and raise trait mindfulness and EF. Further, we expected maintenance of gains during a 1MFU on these outcome measures.

## Methods

### Overall design

This project attained ethics approval at a state university in the eastern part of the USA. Our preregistered randomized trial (NCT04846777 on ClinicalTrials.gov^1^) used a 2 (Intervention: MEMI, SMP) × 3 (Time: pre- to posttreatment, 1MFU) mixed design to test the differential impact of MEMI (versus SMP) on outcomes. Intervention was the between-subject factor, whereas Time was the within-subject factor. A total of 110 participants were recruited (68 MEMIs and 42 SMP). Appendix A in the Online Supplementary Material details the current study’s methods (power analysis, recruitment, compensation, psychometric properties of all measures, EMI measures, data analyses, etc.). Figure S1 in the Online Supplementary Material shows the CONSORT (Consolidated Standards of Reporting Trials) [38] flowchart for participant enrollment and progression.

### Participants

Treatment-seeking participants currently not receiving treatment from a mental health professional were recruited from the psychology subject pool and the local community. Table 1 shows the sociodemographic attributes of study participants. Of the 110 participants randomized to MEMI (*n* = 68) or SMP (*n* = 42), 98 completed the 6-week study protocol by finishing all study visit assessments, and approximately 80% of the app prompts.

**Table 1.** Sociodemographic data of study participants.

	*M*	(SD)	Min	Max
Age (in years)	20.80	(5.41)	18	52
	*n*	(%)		
Gender orientation				
Women	94	(86.67)		
Men	15	(13.63)		
Declined to disclose	1	(0.91)		
Race				
White Caucasian	71	(64.55)		
Asian or Asian American	15	(13.63)		
Hispanic	8	(7.27)		
African American	6	(5.45)		
Another race	2	(1.82)		
Declined to disclose	6	(5.45)		

### Pretreatment clinical interview and screening measure

**Psychiatric diagnoses.** The Anxiety Disorder Interview Schedule-5 (ADIS-5) [39] was a DSM-5-based semi-structured interview [40]. All ADIS-5 interviews were conducted in person or over Zoom^2^ by rigorously trained assessors and video-recorded. Forty percent (*n* = 45) of these video recordings were reviewed and reassessed by another blind rater. Inter-rater agreement was excellent for GAD diagnosis (Cohen’s κ = 1.00) and satisfactory-to-good for other comorbid diagnoses and determination of rule-outs (average κs = 0.75–0.98).

**GAD.** The 14-item Generalized Anxiety Disorder Questionnaire–Fourth version (GAD-Q-IV) [41] that comprised dichotomous (“Yes” or “No” questions) and continuous response formats (e.g., 9-point Likert scale for items measuring interference and distress caused by GAD symptoms) screened for GAD. Also, the GAD-Q-IV measured DSM–Fourth Edition GAD criteria, equivalent to the DSM-5 criteria [40].

### Pre- to posttreatment, and 1-month-follow-up treatment outcomes

**Trait mindfulness.** The 39-item Five Facet Mindfulness Questionnaire (FFMQ) assessed participants’ inclination to engage in five domains of mindfulness: *observing, non-reactivity to inner experiences, non-judgment, describing*, and *acting with awareness.* Participants rated on a 5-point Likert scale (1 = *never or very rarely true*–5 = *very often or always true*). FFMQ total score has shown good convergent validity [42], good discriminant validity from measures of distinct constructs (e.g., psychological well-being) [43], and retest reliability [44]. Cronbach’s αs = 0.76, 0.78, and 0.84 at pretreatment, posttreatment, and 1MFU.

**GAD severity.** GAD severity was assessed with a 16-item GAD-Q-Dimensional measure that paralleled the GAD-Q-IV but consistently included 9-point Likert scale response formats (e.g., 0 = *not at all*–8 = *worry all the time* or 0 = *never*–8 = *almost every day*). The first eight items of the GAD-Q-Dimensional captured trait worry as respondents rated their degree, frequency, controllability, and intensity of worry. The following eight items asked similar questions concerning the past 6 months (αs = 0.90, 0.92, and 0.93).

**Perseverative cognition.** The 45-item Perseverative Cognitions Questionnaire (PCQ) assessed perseverative cognitive traits linked to worry, rumination, and obsessive thoughts. Respondents endorsed items on a 6-point Likert scale (0 = *strongly disagree* to 5 = *strongly agree*). Further, the PCQ-45 comprised six factors: *dwelling on the past*, *expecting the worst*; *lack of controllability*; *thoughts discrepant with ideal self*; *preparing for the future*, and *searching for causes and meanings.* A total score for the PCQ was computed by summing each mean subscale score. The PCQ had strong 2-week retest reliability and discriminant and convergent validity (αs = 0.96, 0.97, and 0.97).

**Inhibition.** The four-condition color-word interference test from the Delis–Kaplan Executive Functioning System [45] measured inhibition. Participants were instructed to read the specific color patches (condition 1 [C1]: color naming) and black-inked color words (C2: word reading) and inhibit the tendency to read the word color and to name the ink color (C3: inhibition) instead. They were asked to alternate between reading the word-color and ink-color of color-words printed in red, blue, or green ink (C4: inhibition/switching). Response times (RTs) were recorded. A composite inhibition score was created by averaging the RTs of C3 and C4, and higher scores denoted worse inhibition (αs = 0.78, 0.84, and 0.87).

### Multi-component mindfulness EMI (MEMI)

For MEMI participants, a standardized video showed the first author conveying principles of evidence-based MBI protocols, such as mindfulness-based stress reduction (MBSR) [46]. MEMI participants were introduced to a definition of mindfulness and asked to concentrate entirely on their current situation and activities. This portion was meant to equip habitual worriers with the skills of *open monitoring* and *attending to small moments.* Next, the video therapist relayed *slowed, rhythmic, diaphragmatic breathing* retraining skills, and subsequently showed how to perform diaphragmatic breathing. Afterward, the video therapist taught MEMI participants *nonjudgmental acceptance.* This component reflected calmness-inducing breathing retraining and mindful observing, non-reactivity, and nonjudgmental acceptance skills delivered in mindfulness-based cognitive therapy (MBCT) [47]. Subsequently, the video therapist informed each MEMI participant of the importance and benefits of practicing mindfulness habitually. Finally, the experimenter implementing the study protocol answered any queries. All experimenters administered the 6-item Credibility and Expectancy Questionnaire (CEQ) [48] and set up the MEMI on each participant’s smartphone (see Appendix B in Online Supplementary Material) after participants indicated that they understood the rationale and mindfulness techniques. Participants received a copy of the MEMI handout via a Qualtrics link and were encouraged to review it regularly.

### Self-monitoring placebo (SMP)

For SMP participants, the standardized video began with the first author *defining self-monitoring* as being highly attentive to one’s cognitions and emotions. The video next proposed to SMP participants that merely *tracking thoughts and recording any related distress* may facilitate thinking in healthier ways. Last, the SMP video relayed the *suggestion that self-monitoring alone could reduce any anxious feelings.* The rationale for the SMP condition was adapted from the treatment rationale used in a recent brief EMI [49]. It was developed to parallel the treatment while eliminating its theorized active therapeutic elements–open monitoring, acceptance, attending to small moments, breathing retraining, and continual mindfulness practice. Thus, it did not mention anything about mindfulness. It did not instruct participants to be more attuned and aware of their current experience (i.e., it focused on monitoring their thoughts and emotions). Also, participants were not asked to focus entirely on their present-moment activities, which would inevitably alter their mood states. As SMP participants were instructed to notice their cognitions and emotions, there was no instruction on accepting their thoughts and feelings as they arose. It also did not provide any breathing retraining instructions. It was not intended to create any form of relaxing sensations that came with abdominal breathing. SMP participants were not asked to practice self-monitoring between the prompts and after treatment ended. The SMP approach thus contrasted the principle that mindfulness was meant to be practiced momentarily and cultivated throughout life. To this end, the SMP was intended to control for credibility and expectancy effects and regression to the mean and prevent inflated effect sizes as would occur with a no-treatment or waitlist control group [50].

The psychoeducation and treatment rationale video for SMP showed the therapist instructing participants to self-monitor by being highly attentive to their cognitions and emotions and observing any distress related to them. Next, like MEMI, all experimenters administered the 6-item CEQ and set up the SMP on their smartphone after each SMP participant showed they understood the rationale and self-monitoring technique (Appendix C in the Online Supplementary Material). Participants received a copy of the SMP handout with no instructions to review it regularly.

### Procedure

During Visit 1, participants first underwent the ADIS-5 clinical interview. Eligible participants then completed initial self-report and performance-based neuropsychological measures in a counterbalanced fashion to rule out order effects. Next, they were randomized to MEMI or SMP with the Microsoft Excel randomization function programmed into Qualtrics with the insertion of the appropriate treatment video played toward the end of Visit 1 after completing all pretreatment assessments. Experimenters/assessors were blinded to treatment conditions, that is, the treatment assignment was concealed from them during all study visits. During Visit 1, the experimenter left the physical room (pre-pandemic) or instructed participants to mute/switch off their Zoom audio and video before they clicked on the Qualtrics link to play the appropriate treatment video (during the pandemic). Participants installed the PACO app programmed with the MEMI or SMP on their smartphones, and the experimenter demonstrated how to use it. Participants were informed that they would be prompted five times daily (about 9 am, noon, 3 pm, 6 pm, and 9 pm) for the next 14 days. The prompts were adjustable based on participants’ schedules. Responses on state depression, anxiety, and mindfulness pre- and post-MEMI or SMP induction had to be keyed in within 2 h of prompting to be valid. The prompts would instruct them to engage in mindfulness or self-monitoring strategies depending on their condition. After 14 days, all participants returned to the laboratory posttreatment and at 1MFU to complete the self-report scales and neuropsychological tests. Participants were compensated by credit hours, money, or a combination of both (Appendix A in the Online Supplementary Material). The research team conducted a seventh-day compliance check and reinvited participants who passed the compliance check.

### Data analyses

Table S1 in the Online Supplementary Material presents the descriptive statistics of all the study variables at various time points. Intent-to-treat analyses were conducted [51]. The analyses included data from 10.71% (*n* = 12) of participants who failed the seventh-day compliance check (i.e., finishing at least 80% of the EMI prompts during the 2-week treatment period). Piecewise hierarchical linear modeling (HLM) analyses were conducted with the *R* package *nlme* [52]. We assumed random intercepts and slopes for all models. Level 1 models within-person changes over time, and Level 2 models between-person factors. We tested the effect of all EMI prompts, examining if treatment led to significantly greater change across the treatment period in pre–post-prompt and the impact of treatment across pre-to-1-month-follow-up (pre-1MFU) while accounting for clustering of repeated measures within persons. Distinct analyses were performed for each outcome. Robust estimators handled non-normality and multivariate outliers without introducing biases to parameter estimates by transforming the data [53] (see Appendix A in the Online Supplementary Material). Between-treatment effect sizes (*d* = 2 *t*/√(*df*)) [54] and within-treatment effect sizes were calculated (*d* = t*√(2/*N*)) [55] to ease interpretation. Between-treatment *d* values of 0.2, 0.5, and 0.8 denoted small, moderate, and large effect sizes, respectively, whereas within-treatment *d* values of 0.5, 0.8, and 1.1 were treated similarly [56]. Readers can refer to the analytic scripts on Open Science Framework (OSF) (https://osf.io/akmcr/).

## Results

### Summary of salient pretreatment characteristics

Table 2 details the salient pretreatment characteristics. Overall participant compliance rate was 89.29% (*n* = 98), and the median number of prompts completed was 63 (range = 0–70). On average, neither treatment credibility nor expectancy significantly differed across conditions (*d* = −0.05–0.19). Table 2 also shows that no significant baseline differences in any outcomes emerged (FFMQ-Total, GAD-Q-Dimensional, PCQ-Total, inhibition) (*d* = −0.240–0.061).

**Table 2.** Summary of salient pretreatment characteristics.

Binary variables	*n*	(%)	Skewness	Kurtosis		
Completion rate	98	(89.091)	2.543	4.547		

Continuous variables	*M*	(SD)	Skewness	Kurtosis	Min	Max
Number of EMA prompts completed	62.500	(17.327)	−2.826	6.987	0	70
Credibility	5.893	(1.465)	−0.085	−0.231	2	9
Expectancy	43.773	(17.560)	0.360	−0.456	10	90

Treatment group differences at pretreatment	β	(SE)	*t*	*d*
Credibility	0.278	(0.288)	0.966	0.186
Expectancy	−0.830	(3.461)	−0.240	−0.046
FFMQ-total	0.028	(0.087)	0.317	0.061
GAD-Q-dimensional	−2.382	(1.888)	−1.262	−0.243
PCQ-total	−0.953	(0.849)	−1.123	−0.216
Inhibition	−0.900	(1.835)	−0.490	−0.094

Note. * p < 0.05; ** p < 0.01; *** p < 0.001.

*Abbreviations: n*, number of participants; %, percentage of total participants; EMA, ecological momentary assessment; *M*, mean; SD, standard deviation; β, beta regression weight; SE, standard error of the regression weight; *t*, *t*-statistic from regression model; *d*, Cohen’s *d* effect size; FFMQ-Total, five factor mindfulness questionnaire-total score; GADQ-IV, generalized anxiety disorder questionnaire–fourth edition; GAD-Q-Dimensional, GADQ-IV-dimensional score; PCQ, perseverative cognitions questionnaire.

### Treatment outcome measures

Table 3 summarizes the HLMs of the between-treatment effects on all trait-level treatment outcome measures at pre–post and pre-1MFU, and Table 4 presents the HLMs of the within-treatment effects.

**Table 3.** Hierarchical linear modeling with random intercepts and slopes for pre- to posttreatment and pre-1MFU time-points, group, and their interaction predicting primary treatment outcomes.

	Pre–post				Pre-1MFU			
	β	(SE)	*t*	*d*	β	(SE)	*t*	*d*
Trait-level Mindfulness (FFMQ-Total)								
Intercept	2.822^***^	(0.069)	41.040	5.534	2.822^***^	(0.069)	41.040	5.534
Condition	0.117	(0.100)	1.176	0.159	0.071	(0.049)	1.457	0.196
Time	0.028	(0.087)	0.317	0.043	0.028	(0.087)	0.317	0.043
Condition ✕ time	0.130	(0.127)	1.025	0.138	0.139*	(0.062)	2.244	0.303
GAD Symptom Severity (GAD-Q-Dimensional)								
Intercept	44.500^***^	(1.484)	29.980	4.043	44.500^***^	(1.484)	29.980	4.043
Condition	−0.548	(2.067)	−0.265	−0.036	−0.393	(0.878)	−0.447	−0.060
Time	−2.382	(1.888)	−1.262	−0.170	−2.382	(1.888)	−1.262	−0.170
Condition ✕ time	−3.008	(2.629)	−1.144	−0.154	−3.173^***^	(1.117)	−2.842	−0.383
Trait-level Perseverative Cognitions (PCQ-Total)								
Intercept	18.021^***^	(0.667)	27.013	3.642	18.021^***^	(0.667)	27.013	3.642
Condition	−1.258	(0.884)	−1.423	−0.192	−0.446	(0.368)	−1.214	−0.164
Time	−0.953	(0.849)	−1.123	−0.151	−0.953	(0.849)	−1.123	−0.151
Condition ✕ time	−0.974	(1.124)	−0.866	−0.117	−1.331^**^	(0.468)	−2.845	−0.384
Inhibition								
Intercept	43.873^***^	(1.365)	32.145	4.334	43.873^***^	(1.365)	32.145	4.334
Condition	−1.879	(0.965)	−1.947	−0.262	−2.276	(0.637)	−3.571	−0.482
Time	−0.372	(1.736)	−0.214	−0.029	−0.372	(1.736)	−0.214	−0.029
Condition ✕ time	−2.528*	(1.228)	−2.059	−0.278	−1.682*	(0.811)	−2.076	−0.280

*Note.* * *p* < 0.05; ^**^ *p* < 0.01; ^***^ *p* < 0.001.

*Abbreviations:* β, beta regression weight; *d*, Cohen’s *d* effect size; 1MFU, 1-month follow-up; FFMQ-Total, five factor mindfulness questionnaire-total score; GADQ-IV, generalized anxiety disorder questionnaire–fourth edition; GAD-Q-Dimensional, GADQ-IV-dimensional score; PCQ, perseverative cognitions questionnaire.

**Table 4.** Within-treatment group simple slope analysis of hierarchical linear modeling with random intercepts.

		Pre–post		Pre-1MFU		Post-1MFU	
Measure	Condition	β	*d*	β	*d*	β	*d*
Trait-level Mindfulness (FFMQ-Total)	MEMI	0.248**	0.521	0.210***	0.953	0.171	0.326
	SMP	0.117	0.275	0.071	0.311	0.024	0.046
GAD Symptom Severity (GADQ-Dimensional)	MEMI	−3.556*	−0.383	−3.567^***^	−0.936	−3.577*	−0.347
	SMP	−0.548	−0.056	−0.393	−0.090	−0.238	−0.023
Trait-level Perseverative Cognitions (PCQ-Total)	MEMI	−2.232^**^	−0.570	−1.777^***^	−0.964	−1.322	−0.286
	SMP	−1.258	−0.295	−0.446	−0.322	0.365	0.081
Inhibition	MEMI	−4.407^***^	−1.053	−3.958^***^	−1.377	−3.509^***^	−0.753
	SMP	−1.879	−0.393	−2.276^**^	−0.760	−2.672^**^	−0.592

*Note.* * *p* < 0.05; ^**^ *p* < 0.01; ^***^ *p* < 0.001.

Abbreviations: β, beta regression weight; *d*, Cohen’s *d* effect size; 1MFU, 1-month follow-up; FFMQ-Total, five factor mindfulness questionnaire – total score; GADQ-IV, generalized anxiety disorder questionnaire – fourth edition; GAD-Q-Dimensional, GADQ-IV-dimensional score; MEMI, mindfulness ecological momentary intervention; PCQ, perseverative cognitions questionnaire; SMP, self-monitoring placebo.

**Figure 1.**
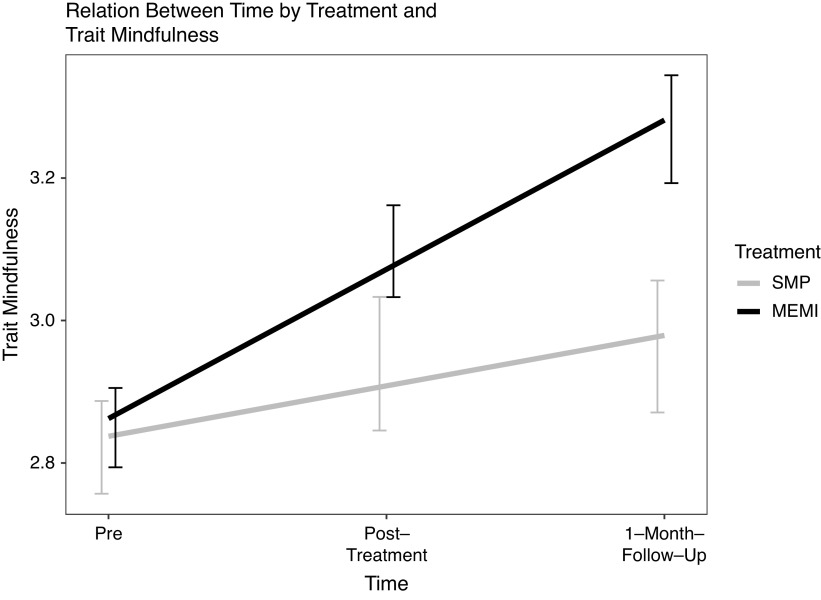
Time by treatment effect on FFMQ. *Note.* FFMQ = five factor mindfulness questionnaire – total score; MEMI = mindfulness ecological momentary intervention; SMP = self-monitoring placebo.

**Trait-level mindfulness.** A significant time ✕ treatment effect emerged during pre-1MFU but not pre–post (*d* = 0.14 versus 0.30) (Figure 1). Nonetheless, at pre–post, simple slope analyses showed a significant increase in trait mindfulness in MEMI (versus SMP) (*d* = 0.52 versus 0.28). At pre-1MFU, there was a greater increase in trait mindfulness in the MEMI (versus SMP) (*d* = 0.95 versus 0.31) (Table 4).

**Figure 2.**
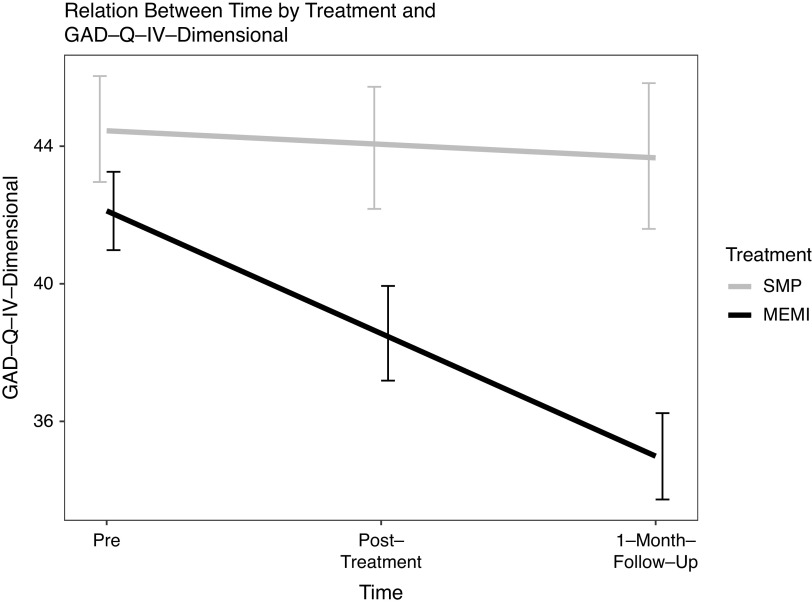
Time by treatment effect on GAD dimensional severity. *Note.* GAD-Q-IV-Dimensional = generalized anxiety disorder-questionnaire-fourth edition (GADQ-IV)-dimensional score; MEMI = mindfulness ecological momentary intervention; SMP = self-monitoring placebo.

**GAD symptom severity.** A significant time ✕ treatment effect occurred at pre-1MFU but not pre–post (*d* = −0.38 versus −0.15) (Figure 2). However, at pre- to posttreatment, simple slope analyses indicated a significant decrease in GAD symptom severity in MEMI but not SMP (*d* = −0.38 versus −0.056) (Table 4). Also, during pre-1MFU, a substantial reduction in GAD symptom severity occurred in MEMI (versus SMP) (*d* = −0.93 versus 0.31).

**Figure 3.**
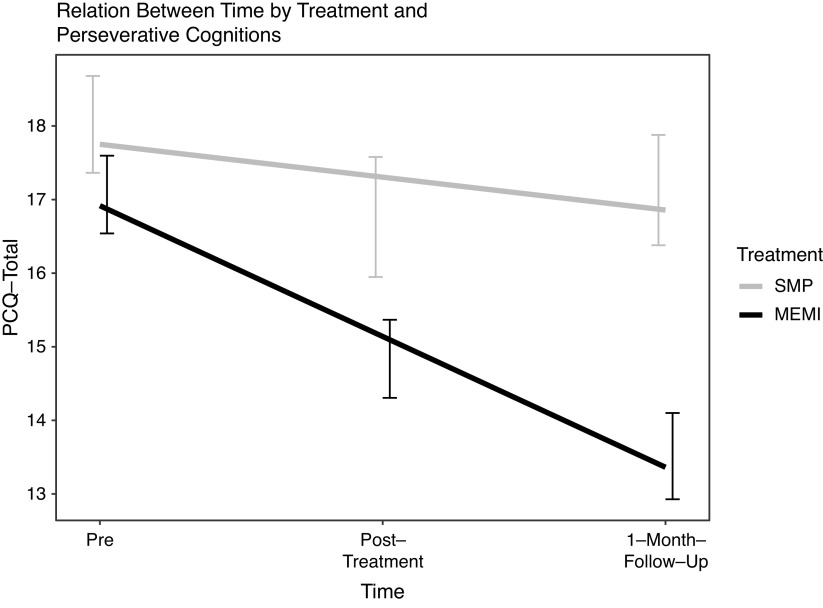
Time by treatment effect on PCQ-total. *Note.* PCQ = perseverative cognitions questionnaire; MEMI = mindfulness ecological momentary intervention; SMP = self-monitoring placebo.

**Trait-level perseverative cognitions.** A significant time ✕ treatment effect emerged during pre-1MFU but not pre- to posttreatment (*d* = −0.38 versus −0.12) (Figure 3). Nonetheless, during pre-to posttreatment, a considerable reduction in perseverative cognitions was observed for MEMI but not SMP (*d* = −0.57 versus −0.29; Table 4). Likewise, during pre-1MFU, perseverative cognitions significantly decreased in MEMI but not SMP (*d* = −0.96 versus −0.32).

**Figure 4.**
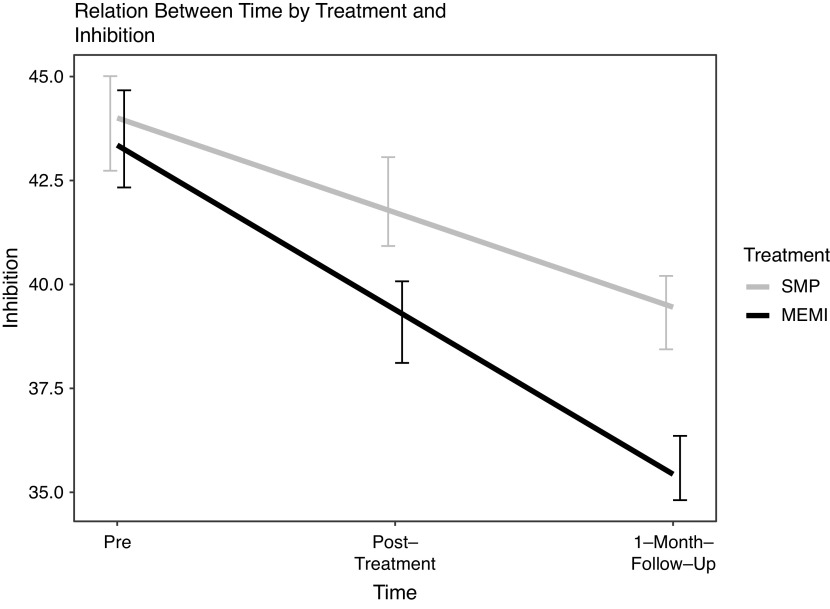
Time by treatment effect on inhibition. *Note.* MEMI = mindfulness ecological momentary intervention; SMP = self-monitoring placebo.

**Inhibition.** A significant time ✕ treatment effect on inhibition RT was observed at pre- to posttreatment (*d* = −0.28) and pre-1MFU (*d* = −0.28) (Figure 4). At pre- to posttreatment, a significant reduction in inhibition RT was produced by MEMI but not SMP (*d* = –1.05 versus –0.39). During pre-1MFU, the significant decrease in inhibition RT was significantly larger in MEMI (versus SMP) (*d* = −1.38 versus −0.76) (Table 4).

Table S2 of the Online Supplementary Material shows the maintenance of treatment gains on the above four measures.

### State measures of anxiety, depression, and mindfulness

Across the 14-day pre- to posttreatment, significant induction ✕ treatment effects were found for state anxiety (*d* = −0.50), state depression (*d* = −0.76), and state mindfulness (*d* = 1.13). From pre- to posttreatment, the inductions showed a larger reduction in state anxiety for MEMI (versus SMP) (*d* = −1.17 versus −0.49). Whereas pre–post-induction state depression was significantly reduced in MEMI, it unexpectedly substantially increased in SMP (*d* = −0.84 versus 0.68). Similarly, whereas pre–post-induction state mindfulness significantly increased in MEMI, it unexpectedly notably declined in SMP (*d* = 1.52 versus −0.97).^3^

## Discussion

Results showed that a 2-week MEMI versus SMP led to a significantly greater increase in state mindfulness and greater reductions in state anxiety and state depression pre- to posttreatment. Simultaneously, MEMI was significantly better than SMP in decreasing GAD severity and repetitive negative thinking and enhancing trait mindfulness at pre-1MFU, but there was no differential treatment efficacy on these measures at pre–post treatment. We also found significant between-treatment effects favoring MEMI on stronger inhibition at pre- to posttreatment and pre-1MFU. Although some within-treatment significant effect sizes were moderate-to-large, the between-treatment effect sizes were generally small, signaling that any present enthusiasm surrounding brief digitally-delivered MBIs should be tempered pending further research.

Our treatment effects were comparable to the small yet significant effect sizes observed in other brief app and Internet-based psychotherapy trials for GAD [49, 57]. Moreover, data pooled across mainly self-directed CBT and relaxation training-focused EMI RCTs for GAD and other anxiety disorders similarly showed small-to-moderate effects in reducing anxiety severity [58, 59]. Our findings were additionally consistent with small yet notable effect sizes of meditation apps (versus active and non-specific controls) targeting anxiety and depressive symptoms [60]. Further, our high retention and compliance rates were noteworthy, starkly contrasting most mHealth platforms that demonstrated low degrees of engagement and completion [61]. We build on prior literature [62] by extending research that an 8-week evidence-based manualized face-to-face and web-based MBI (e.g., MBSR) [63, 64] *and* a brief MEMI could enhance trait mindfulness, albeit with smaller effect sizes.

Why were there substantially larger pre-1MFU than pre- to posttreatment reductions in GAD severity and perseverative cognition tendencies in MEMI than SMP? Perhaps the MEMI (versus SMP) conferred more robust improvements in observational skills and engagement in moment-to-moment awareness, acceptance, and receptivity to physical sensations, thoughts, and experiences [65]. Moreover, MEMI, unlike SMP, offered opportunities to exercise mindfulness regularly in everyday life and emphasized the importance of cultivating those skills throughout life. Plausibly, MEMI participants needed to regularly practice various mindfulness skills beyond the 2-week intervention to reap notable benefits. Exercising mindfulness repeatedly in real-time persistently via the MEMI might have assisted habitual worriers in identifying moments where practicing slowed breathing, non-judgment, and non-reactivity strategies were required. The continual practice could avert unhelpful thoughts from perpetuating and NA states from emerging, perhaps via increased engagement in values-oriented, goal-directed activities in the here and now, as instructed by the MEMI.

What are plausible change mechanisms via which MEMI outperformed SMP in reducing GAD severity, perseverative cognitions, and promoting trait mindfulness at pre-1MFU? Practicing mindfulness moment-to-moment might have enhanced present-mindedness and metacognitive skills (i.e., non-reactively observing emotions and thoughts in real-time) versus being overly preoccupied while brooding or worrying [66]. Prior trials indeed showed that decrements in various repetitive thoughts mediated the impact of MBI on anxiety, depression, and distress [67]. Other conceivable accounts are that MEMI disrupted reactive repetitive thoughts by enhancing one’s curiosity about various experiences and feelings (including NA) and shifting focus toward rewarding activities [68]. These ideas are consistent with evidence that therapist-led MBIs raised trait-like specificity of life goals and perseverance [69] and enhanced state-level positive emotions, gratitude, and constructive responses to pleasant everyday activities in persistently depressed adults [70, 71]. Future EMI studies should heed calls to include process measures [72] and examine therapeutic processes through an idiographic lens [73].

Consistent with *mindfulness-cognitive enhancement models* [14, 74], MEMI (versus SMP) had a more significant effect on enhancing inhibition at pre–post and pre-1MFU. Such data are congruous with evidence that exercising mindfulness breathing techniques for 3 weeks could improve inhibition and conflict monitoring in healthy controls [75] and patients with psychosis [76]. The possibility that MEMI optimized attention, cognitive control, and EF-related brain pathways [77, 78] could explain these findings [79]. These hypotheses await empirical evaluation.

Some unanticipated impacts of SMP emerged during pre- to posttreatment. Whereas MEMI reduced state depression and anxiety and increased state mindfulness, SMP raised state depression and decreased state mindfulness across pre- to posttreatment. Such results were inconsistent with evidence that self-monitoring alone could positively affect worry severity [49] and state NA [80]. Future research could test the notion that self-monitoring could, at times, lead to increased distress in the short term as participants become more acutely aware of their emotions. Also, the SMP instructions that explicitly requested participants to focus on distress related to their thoughts and feelings might contribute to these results.

Findings need to be construed in light of the strengths and limitations herein. First, the 2-week treatment duration might be insufficient to produce more immediate improvements in trait mindfulness, GAD severity, perseverative cognitions, and inhibition; nonetheless, the findings appeared more promising for habitual worriers during pre-1MFU. Second, our study did not include measures to evaluate how MEMI participants continued practicing mindfulness skills posttreatment to 1MFU. Future studies should thus consider whether unceasing mindfulness practices, even without repeated instructions via the MEMI, might have contributed to any differential treatment efficacy at follow-up. Also, our study inferences might not generalize beyond primarily White females, highlighting the importance of future digital mental health trials to recruit a more diverse sample. In addition, despite randomization, the intervention and control groups had uneven sample sizes. Study strengths included the gold standard RCT design with active control, high compliance rate, recruitment of a well-powered clinical sample, and inclusion of a 1MFU assessment. Also, our attrition rate of 11% was lower than the average of 24 to 50% in smartphone-delivered RCTs [81, 82]. Our low attrition rate might be due to the current study’s pro-rated design of the reimbursement schedule.

If our pattern of findings is replicated, some clinical implications merit consideration. Promoting the use of MEMI in treatment for GAD may bridge the chasm between the therapist’s office and chronic worriers’ everyday life. As clients continually use mindfulness skills, new helpful action repertoires and mindsets might replace old limiting habits, such as avoiding shifts from positive or neutral to negative states [83]. Therapists can also emphasize that these practices might assist with alleviating anxiety, reducing GAD symptoms and perseverative thoughts, and improving EF. Future trials could examine how individualized feedback [84], event-contingent triggers, and passive sensors [85] could increase the efficacy of MEMI in targeting GAD (just-in-time adaptive interventions) [86].

## Data Availability

All authors will make the data used in this publication available upon request. Moreover, the *R* input syntax has been uploaded to Open Science Framework (https://osf.io/akmcr/) and a pre-print to PsyArXiv (https://psyarxiv.com/t3egn).

## Notes

1 We could not reach our target of recruiting 150 participants per intervention arm due to COVID-19 restrictions and funding limitations.

2 The study protocol has been executed over Zoom since the start of the COVID-19 pandemic.

3 All findings remained similar and statistically significant after accounting for the data collection period (0 = *pre-COVID-19 pandemic before March 2020*, 1 = *March 2020 to January 2021*).

## Supplementary material

For supplementary material accompanying this paper visit http://doi.org/10.1192/j.eurpsy.2023.2. click here to view supplementary material
