# Supplementary material for: A randomized controlled trial of a 14-day mindfulness ecological momentary intervention (MEMI) for generalized anxiety disorder
Source: Eur Psychiatry. 2023 Jan 16;66(1):e12. doi: 10.1192/j.eurpsy.2023.2 (PMC9970156; doi:10.1192/j.eurpsy.2023.2)
Supplement: Supplementary file 1 [file S0924933823000020sup001.docx]

# Online Supplementary Materials (OSM)

Table S1

*Descriptive Statistics of Study Variables*

|  | *M* | (SD) | Min | Max | Skewness | Kurtosis |
| --- | --- | --- | --- | --- | --- | --- |
| **Pre-treatment: SMP** | |  |  |  |  |  |
| FFMQ-Total | 2.822 | (0.422) | 2.093 | 3.675 | 1.582 | -0.012 |
| GADQ-IV-Dimensional | 44.500 | (9.981) | 16.000 | 62.000 | -0.700 | 0.635 |
| PCQ-Total | 18.021 | (4.267) | 5.197 | 26.816 | -0.389 | 0.573 |
| Inhibition | 47.447 | (7.822) | 32.500 | 80.035 | 1.706 | 5.496 |
| **Pre-treatment: MEMI** | |  |  |  |  |  |
| FFMQ-Total | 2.850 | (0.460) | 2.839 | 2.836 | 0.432 | 1.843 |
| GADQ-IV-Dimensional | 42.118 | (9.391) | 13.000 | 60.000 | -0.426 | 0.248 |
| PCQ-Total | 17.068 | (4.358) | 2.143 | 26.519 | -0.663 | 0.895 |
| Inhibition | 46.548 | (10.172) | 20.095 | 82.500 | 0.814 | 1.890 |
| **Post-treatment: SMP** | |  |  |  |  |  |
| FFMQ-Total | 2.939 | (0.608) | 2.993 | 2.971 | 0.442 | 1.500 |
| GADQ-IV-Dimensional | 43.952 | (11.508) | 22.000 | 72.000 | 0.647 | 0.496 |
| PCQ-Total | 16.764 | (5.284) | 3.656 | 26.840 | -0.234 | -0.254 |
| Inhibition | 43.050 | (6.327) | 22.885 | 59.525 | -0.060 | 2.015 |
| **Post-treatment: MEMI** | |  |  |  |  |  |
| FFMQ-Total | 3.097 | (0.532) | 3.082 | 3.091 | 0.601 | 2.082 |
| GADQ-IV-Dimensional | 38.562 | (11.348) | 11.200 | 62.000 | 0.033 | -0.768 |
| PCQ-Total | 14.836 | (4.375) | 6.000 | 26.232 | 0.319 | -0.339 |
| Inhibition | 40.829 | (8.276) | 17.300 | 65.000 | 0.684 | 1.873 |
| **1-Month Follow-Up: SMP** | | |  |  |  |  |
| FFMQ-Total | 2.964 | (0.600) | 3.057 | 3.008 | 0.432 | 1.560 |
| GADQ-IV-Dimensional | 43.714 | (13.731) | 14.000 | 72.000 | -0.120 | -0.012 |
| PCQ-Total | 17.129 | (4.858) | 6.508 | 26.312 | -0.024 | -0.641 |
| Inhibition | 41.063 | (4.718) | 30.415 | 54.845 | 0.148 | 0.740 |
| **1-Month Follow-Up: MEMI** | | |  |  |  |  |
| FFMQ-Total | 3.269 | (0.624) | 3.293 | 3.256 | 0.530 | 1.832 |
| GADQ-Dims | 34.985 | (10.353) | 12.000 | 61.000 | 0.157 | -0.096 |
| PCQ-Total | 13.514 | (4.833) | 4.900 | 25.773 | 0.475 | -0.348 |
| Inhibition | 37.966 | (6.755) | 25.310 | 75.155 | 2.556 | 11.985 |

*Note.* FFMQ=five facet mindfulness questionnaire; GADQ-IV=generalized anxiety disorder questionnaire–fourth edition–dimensional score; MEMI=mindfulness ecological momentary intervention; PCQ=perseverative cognitions questionnaire; SMP=self-monitoring placebo; VFS=verbal fluency score.

Table S2

*Hierarchical Linear Modeling with Random Intercepts for Post-1MFU Time-points, Group, and Their Interaction Predicting Primary Treatment Outcomes*

| Outcome | β | (*SE*) | *t* | *d* |
| --- | --- | --- | --- | --- |
| FFMQ-Total |  |  |  |  |
| Intercept | 2.915^***^ | (0.180) | 16.210 | 2.186 |
| Condition | 0.024 | (0.115) | 0.211 | 0.028 |
| Time | 0.011 | (0.229) | 0.047 | 0.006 |
| Condition ✕ Time | 0.147 | (0.146) | 1.008 | 0.136 |
| GADQ-IV-Dimensional |  |  |  |  |
| Intercept | 44.190^***^ | (3.640) | 12.140 | 1.637 |
| Group | -0.238 | (2.273) | -0.105 | -0.014 |
| Time | -2.052 | (4.630) | -0.443 | -0.060 |
| Group ✕ Time | -3.338 | (2.891) | -1.155 | -0.156 |
| PCQ-Total |  |  |  |  |
| Intercept | 16.398^***^ | (1.587) | 10.334 | 1.393 |
| Group | 0.365 | (1.002) | 0.365 | 0.049 |
| Time | -0.240 | (2.018) | -0.119 | -0.016 |
| Group ✕ Time | -1.688 | (1.274) | -1.325 | -0.179 |
| Inhibition |  |  |  |  |
| Intercept | 44.666^***^ | (1.982) | 22.534 | 3.039 |
| Group | -2.672^**^ | (1.005) | -2.658 | -0.358 |
| Time | -2.064 | (2.521) | -0.819 | -0.110 |
| Group ✕ Time | -0.837 | (1.279) | -0.654 | -0.088 |

*Note.* ^*^ *p* < .05; ^**^ *p* < .01; ^***^ *p* < .001.

β=regression weight; *d=*Cohen's *d* effect size; 1MFU=1-month follow-up; GADQ-IV=generalized anxiety disorder questionnaire–fourth edition–dimensional score; PCQ=perseverative cognitions questionnaire.

Figure S1

*CONSORT Flowchart of Participant Recruitment and Progress*

*Note.* CONSORT=Consolidated Standards of Reporting Trials; MEMI=mindfulness ecological momentary intervention; SMP=self-monitoring placebo.

# Appendix A

**Overall Design**

Based on an *a priori* Monte Carlo power analysis (Arend & Schafer, 2019; Magnusson, 2018), the current study had 80.64%–92.14% power to detect a significant Treatment × Time interaction with a small-to-moderate effect size of Cohen's *d*=0.25.

**Participants**

The study was advertised on StudyFinder. Participants had to meet the criteria for GAD based on the Diagnostic and Statistical Manual–Fifth Edition (DSM-5; American Psychiatric Association, 2013). First, potential participants were screened using the 14-item Generalized Anxiety Disorder Questionnaire–Fourth version (GAD-Q-IV; Newman et al., 2002). Those who had GAD indexed by the DSM-5 algorithm on the GAD-Q-IV self-report (Moore et al., 2013; Newman et al., 2002) were invited to undergo the Anxiety Disorder Interview Schedule-5 (ADIS-5; Brown & Barlow, 2014) to ascertain GAD and other psychiatric diagnoses. Also, participants were at least 18 years of age, owned an iPhone or Android phone, and provided informed consent. Exclusion criteria included the presence of suicidality, mania, psychosis, or substance use disorders.

In addition, participants were reimbursed up to $30, 6 subject pool credits, or a mixture of both forms of compensation to fulfill course requirements pro-rated based on their degree of participation and assessment completion. Subject pool participants only receiving credits received 3.5 subject pool credit hours for completion of the first part of the study (before the eighth day of intervention use). Participants who sufficiently completed study tasks up to the seventh-day compliance check were invited to finish the second part of the study and received an additional 2.5 subject pool credit hours.

Participants who received subject pool credit *and* monetary compensation received the remaining course credit hours they needed to reach their required six subject pool hours credit. After receiving these credit hours, they received up to $5.00 for each additional hour spent in the study. The amount they received was the percentage of study tasks and responses to smartphone prompts multiplied by $5.00 for each remaining hour of participation based on this equation to calculate their final compensation amount: [credit hours + (% task completion x ($5.00 x their remaining hours of participation))]. These participants could not start receiving money until they completed all their needed credit hours (for at least 6 hours). They received 3.5 subject pool credit hours for completing the first part of the study (before the eighth day of intervention use). If participants sufficiently completed study tasks up to the seventh-day compliance check, they were invited to finish the second part of the study and receive an additional 2.5 subject pool credit hours (up to their total of 6) OR their task completion percentage multiplied by $5.00 for each remaining hour. For example, if they only needed 2 more credit hours to reach their total of 6, then completed 90% of tasks/prompts in the study's last 4 hours, they received 2 credit hours plus .90 x ($5 x 4 hours) = $18.00.

Participants receiving only money received up to $30.00, i.e., $30.00 times the percentage of study tasks and responses to smartphone prompts that they completed. Stated differently, they received $5.00 per hour of participation multiplied by the percentage of study tasks/prompts they completed. The maximum was $30.00 ($5 x 6 hours). They received up to $17.50 for 100% completion of the first part of the study (before the eighth day of intervention use). If they sufficiently completed study tasks up to the seventh-day compliance check, they were invited to finish the second part of the study. In this second part of the study, they had the opportunity to receive an additional $12.50 with 100% completion. For example, if they completed 93% of study tasks and completed all 6 hours of participation, they received .93 x ($5.00 x 6hr) = $27.90.

Participants who decided to withdraw during the study received subject pool credits, money, or a mixture of both based on their degree of study participation. If they were a subject pool participant requiring only credits, we rounded up the amount of subject pool credit hours awarded to them. For example, consider a subject pool participant who required 3 more subject pool credits and decided to withdraw after completing 45% of the study protocol. Instead of awarding 2.7 credits (45% of 6 credits), we rounded up that figure and awarded 3 credits instead. If they were a participant receiving monetary compensation and decided to withdraw after 3 hours of participation and completed 90% of the study tasks/prompts, they received .90 x (3/6) x ($30.00) = $13.50.

**Pre-Treatment Clinical Interview and Screening Measure**

**Psychiatric diagnoses.** Forty percent (*n*=45) of these video recordings were reviewed and re-assessed by another blind rater. Inter-rater agreement was excellent for GAD diagnosis (Cohen's κ=1.00) and satisfactory-to-good for other comorbid diagnoses and determination of rule-outs (average κs=0.75–0.98). The ADIS-5 demonstrated high convergence with the Structured Clinical Interview for DSM (SCID; Spitzer et al., 1994) for GAD, major depressive disorder, and other disorders (Shankman et al., 2018), with an excellent inter-rater agreement (κ=.88 to 1.00) (Wade et al., 2022) and strong two-week retest reliability (Rutter & Brown, 2015). The ADIS-5 also showed good convergent and discriminant validity (Gordon & Heimberg, 2011).

**GAD.** The 14-item GAD-Q-IV (Newman et al., 2002) DSM-5 GAD algorithm showed good sensitivity (78-81%) and specificity (86-97%) compared to a clinical interview (Moore et al., 2014; Newman et al., 2002). Convergent and discriminant validity were evidenced by large relations with trait anxiety and worry and small associations with unique constructs (e.g., depression; Newman et al., 2002). It also showed good two-week retest reliability (Newman et al., 2002) and strong internal consistency. In the present study, Cronbach’s αs=.80, .89, and .91 for GAD-Q-IV (total possible score=0–14) at baseline, post-treatment, and 1MFU.

**Pre-, Post-Treatment, and 1-Month-Follow-Up Self-Report Measures**

**Trait Mindfulness.** FFMQ observing involves noticing and paying attention to environmental (e.g., sounds, sights, smells) and internal events (e.g., texture, physical sensations, thoughts) (8 items; e.g., "When I'm walking, I deliberately notice the sensations of my body moving."). Nonjudgment comprises adopting a nonevaluative attitude toward thoughts and emotions (8 items; e.g., of a reverse-scored item, "I criticize myself for having irrational or inappropriate emotions."). Non-reactivity to inner experiences refers to the tendency to permit the ebb-and-flow of thoughts and emotions without being distracted by or overly focused on them (7 items; e.g., "I perceive my feelings and emotions without having to react to them."). Describing is labeling inner experiences with words (8 items; e.g., "I'm good at finding words to describe my feelings."). Acting with awareness entails attentively engaging in present-moment tasks rather than doing things absent-mindedly or on autopilot (8 items; e.g., of a reverse-scored item.").

**GAD Severity.** The psychometric properties of the GAD Severity scale were ascertained with a dataset of 883 subject pool screen participants with high score variability. Convergent and discriminant validity were evidenced by moderate-to-large relations with the GAD-Q-IV (*r*=.87) and small relations with trait rumination (*r*=.44) and spider phobia (*r*=.11). A confirmatory factor analysis showed that it had excellent model fit (χ^2^(*df*=104)=96.85, *p*=.678, Confirmatory Fit Index=1.000, Tucker-Lewis Index=1.000, Root Mean Square Error of Approximation=.000, 95% CI [.000, 014], and Standardized Root Mean Square Residual=.029).

**Perseverative cognition.** The 45-item PCQ comprised six factors: *dwelling on the past* (DP; 14 items: e.g., "I cannot help but rehash past events in my mind"); *expecting the worst* (EW; 4 items: e.g., "I usually expect the worst in ambiguous situations"); *lack of controllability* (LC; 5 items: e.g., "I am surprised by how little control I have over certain thoughts"); *thoughts discrepant with ideal self* (DT; 11 items: e.g., "I feel appalled by some of my thoughts"); *preparing for the future* (PF; 7 items: e.g., "I repeatedly think about a current problem in order to avoid it"); *searching for causes and meanings* (SC; 4 items: e.g., "I repeatedly think about my feelings to discover if they have some deeper meaning").

**Pre-, Post-Treatment, and 1-Month-Follow-Up Behavioral EF Measures**

**Inhibition.** The color-word interference test had strong retest reliability (*r*=.62–.76), good convergent with scores on other inhibition measures, and discriminant validity with scores on measures of other constructs (Homack et al., 2005; Martínez-Loredo et al., 2017).

**EMI Self-Report Measures Across 14-Days from Pre-Post Treatment**

Ecological momentary assessments of state symptoms and mindfulness were administered before and after exposure to the MEMI or SMP instructions during each prompt.

**State depression, anxiety, and mindfulness.** Participants rated on two 9-point Likert scales (1=*Not at All* to 9=*Extremely*) their degree of state depression ("To what degree do you feel depressed right now?"), anxiety ("To what degree do you feel keyed up or on edge right now?"), and mindfulness ("To what extent are you experiencing the present moment fully?") before and after practicing the skills based on their assigned treatment condition.

**Treatment Credibility and Expectancy Questionnaire (CEQ; Devilly & Borkovec, 2000).** The 6-item CEQ measured the degree to which participants believed the treatment was credible (e.g., "At this point, how logical does the therapy offered to you seem?"; 1=*not at all logical* to 9=*very logical*) and would substantially change symptoms (e.g., "By the end of the therapy period, how much improvement in your symptoms do you think will occur?"; 0%–100%). The CEQ showed strong retest reliability (Devilly & Borkovec, 2000) and high internal consistency (αs=0.86 and 0.88 for credibility and expectancy herein, respectively). On average, neither treatment credibility (MEMI: 6.00 (1.39); SMP: 5.72 (1.58), *p*=.336, *d*=0.19) nor expectancy (MEMI: 43.46 (17.33); SMP: 44.29 (18.13), *p*=.310, *d*=0.20) significantly differed across conditions.

**Multi-Component Mindfulness EMI (MEMI)**

Appendix B displays screenshots of MEMI prompts. During each EMI prompt, MEMI participants were first instructed to engage in *slowed, steady, rhythmic breathing*, "Pay attention to your breathing. Breathe in a slow, steady, and rhythmic manner. Stay focused on the sensations of the air coming into your lungs and then letting it out. Click 'Continue'". Then, they were instructed to practice *open monitoring* and *acceptance*, "As you're breathing, observe your experience as it is. Let go of judgments that do not serve you. Focus your attention in the here-and-now. Click 'Continue.'" Last, they were instructed to *attend to small moments*: "Attend to the small moments right now (e.g., reading a chapter, having a cool glass of water) as that is where enjoyment, peace, and serenity in life happen. Click 'Okay' to continue."

**Self-Monitoring Placebo (SMP)**

The psychoeducation and treatment rationale video for SMP showed the therapist reading verbatim the script that instructed participants to self-monitor by being highly attentive to their cognitions and emotions and observing any distress related to them. Next, like MEMI, all experimenters administered the 6-item CEQ after each SMP participant showed they understood the rationale and self-monitoring technique. Following this, the experimenter provided a copy of the handout of the self-monitoring placebo but did not instruct them to review it regularly. Appendix C shows screenshots of the EMI prompts for the SMP condition. During each EMI prompt, SMP participants were asked to observe their thoughts, "Notice your thoughts and how distressing they may be. Click 'Okay' to continue."

## Data Analyses

In total, 3.55% of the data was missing. Consistent with an intent-to-treat approach, missing data were managed using multiple imputation (Graham, 2009) by aggregating data across 100 imputed datasets, each with 10 iterations. Lower baseline WM and non-White (compared to White) substantially predicted attrition. Also, MEMI, but not SMP, participants significantly more likely to drop out were females (vs. males or unknown gender) and those with higher baseline GAD-Q-Dimensional severity (refer to Appendix D). Thus, we included these significant predictors of attrition as auxiliary variables to improve the precision of the multiple imputation model (Enders et al., 2020).

In addition, we detected no univariate outliers, and most study variables had acceptable skewness (within±3) and kurtosis (within±8) values. Furthermore, we determined that sociodemographic variables (age, gender, ethnicity) and study enrollment time (before or during the COVID-19 pandemic) did not significantly predict primary and secondary outcomes (*p* values ranged from .072 to .917). Therefore, we did not adjust for these variables as covariates in our series of multilevel models.

# Appendix B

## Screenshots for Treatment Condition


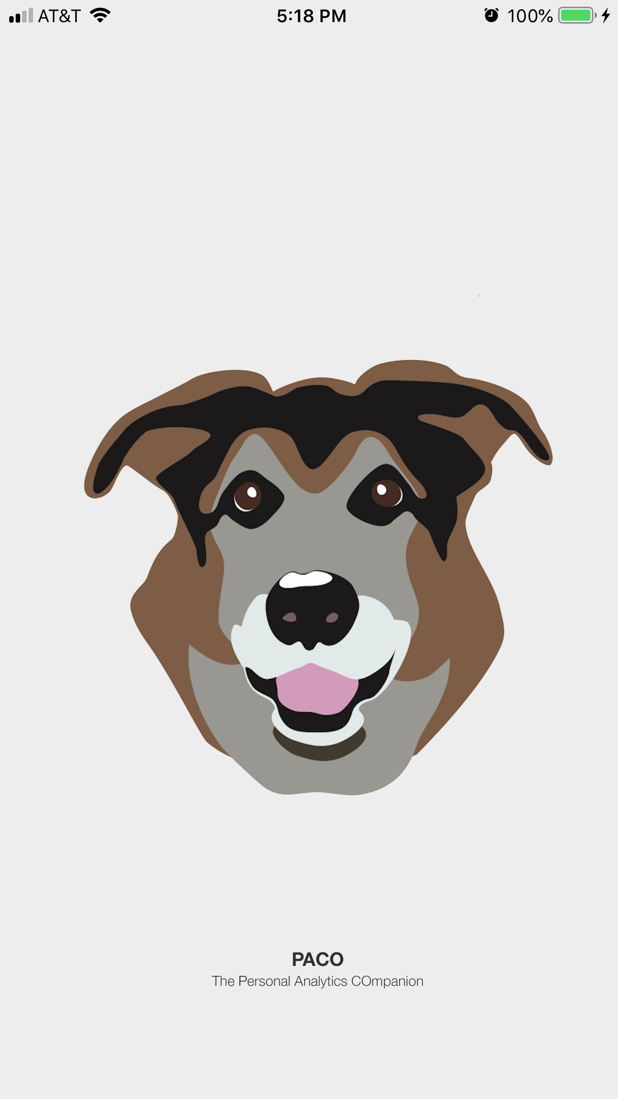

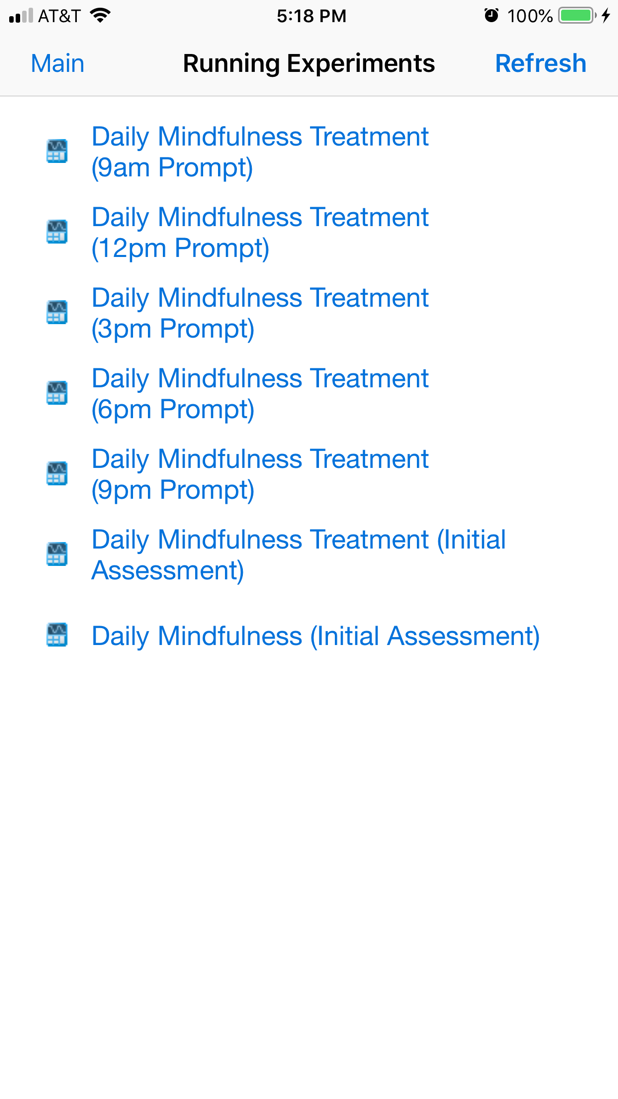


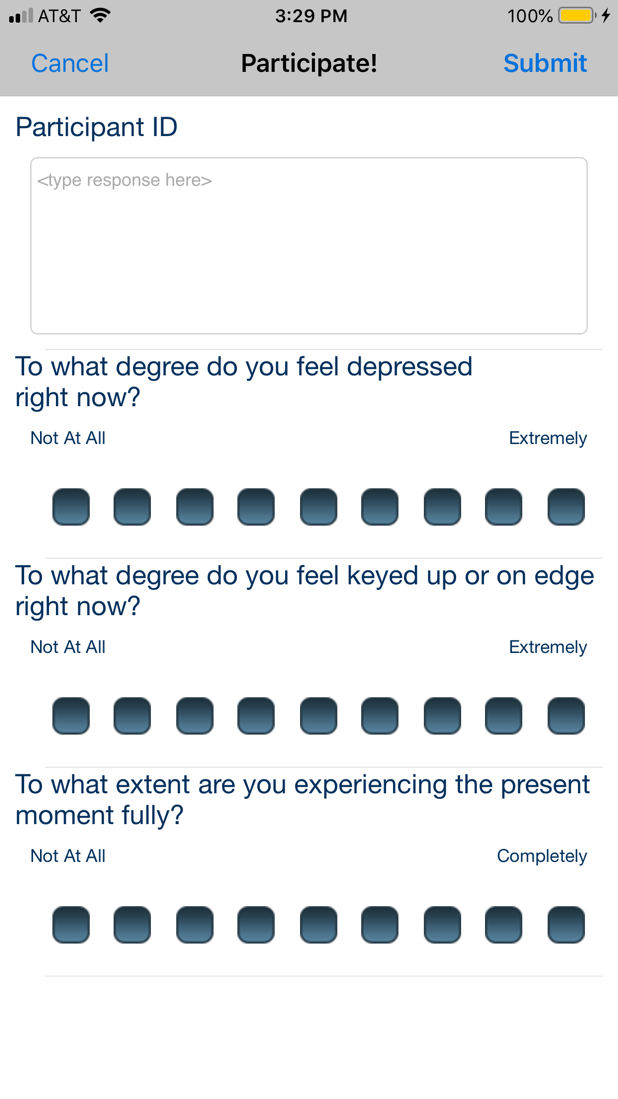

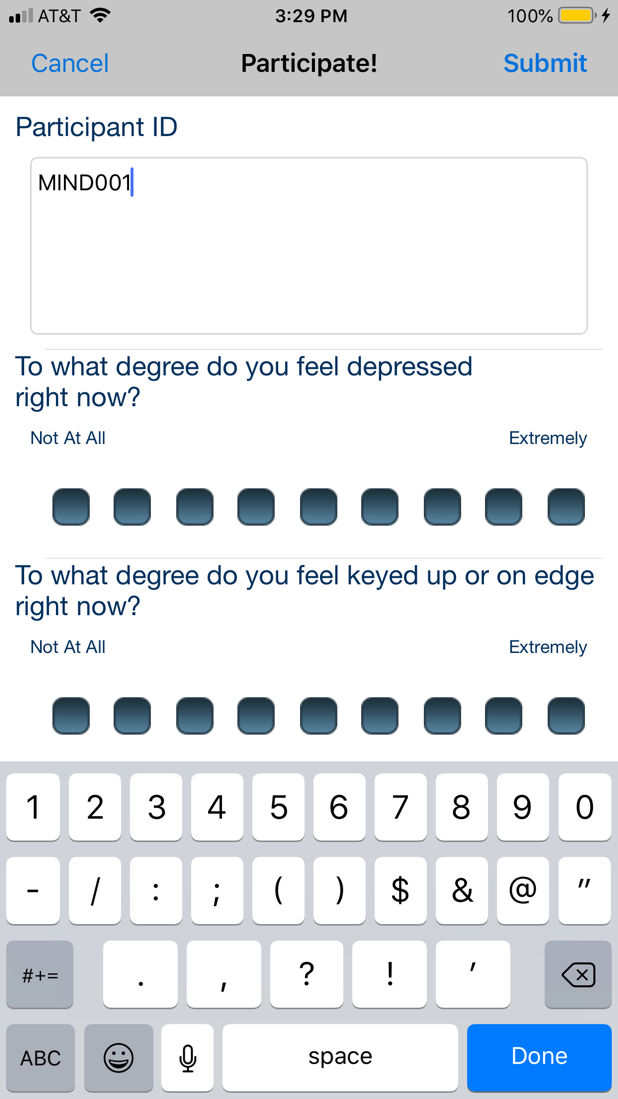


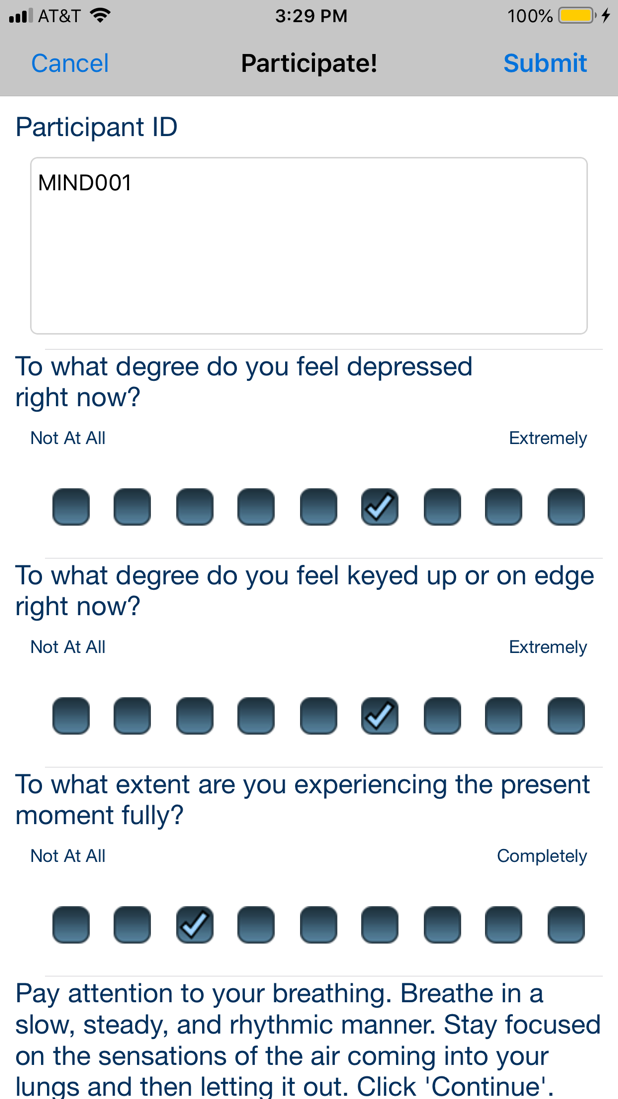

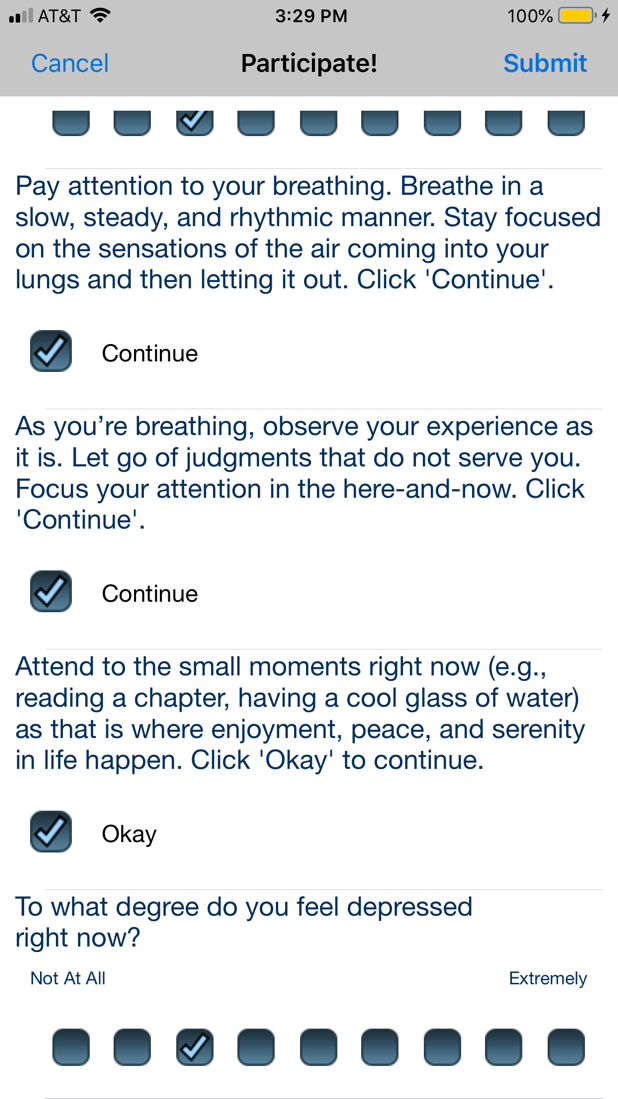


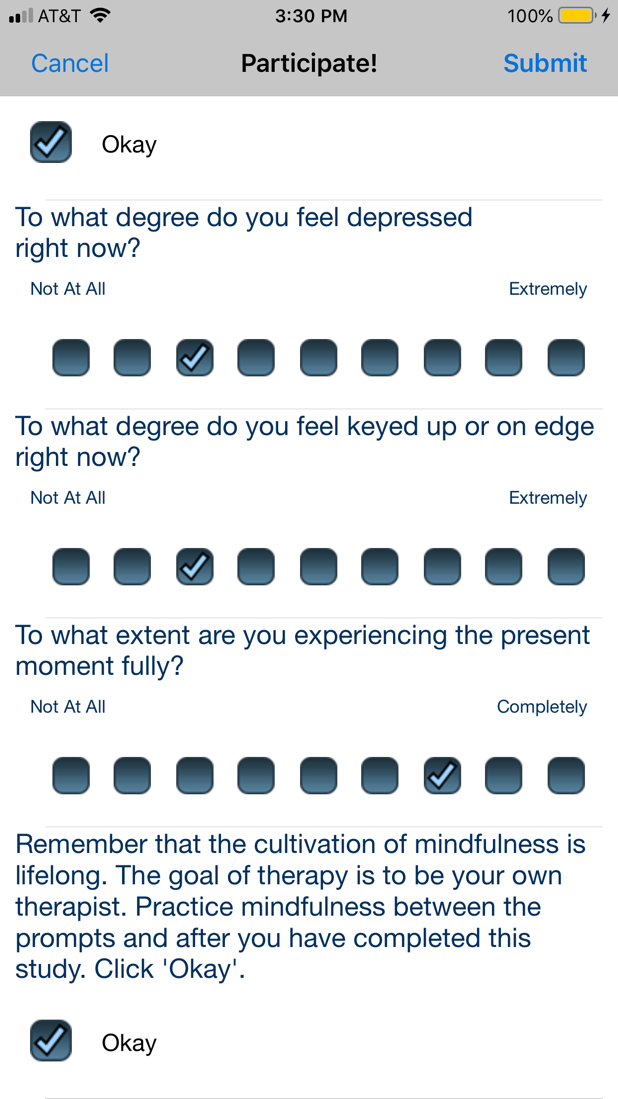

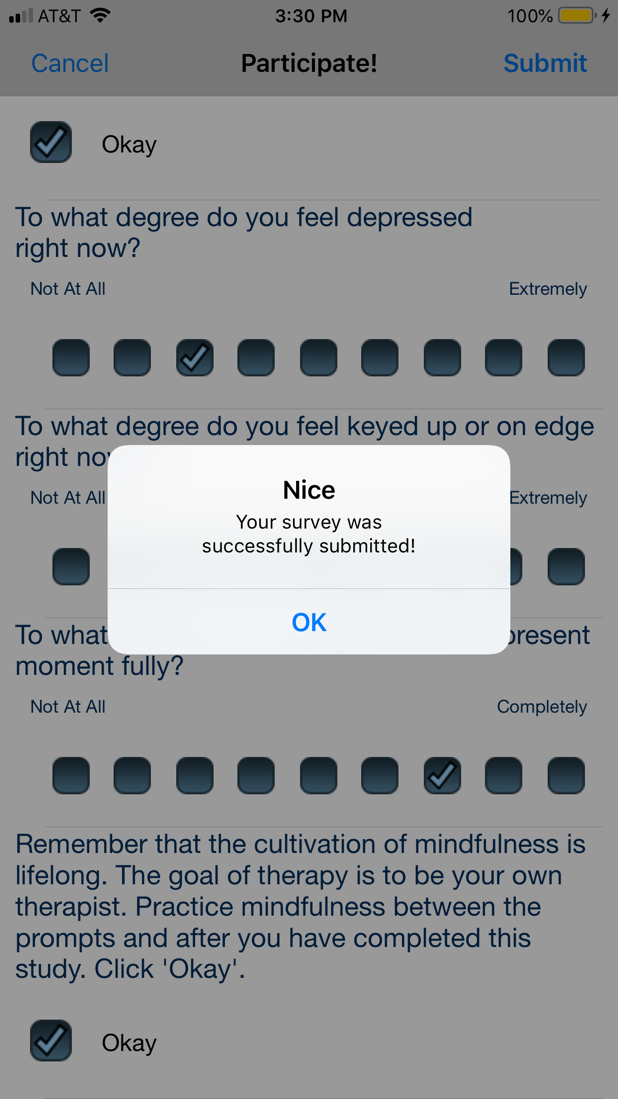


# Appendix C

## Screenshots for Self-Monitoring Placebo Condition


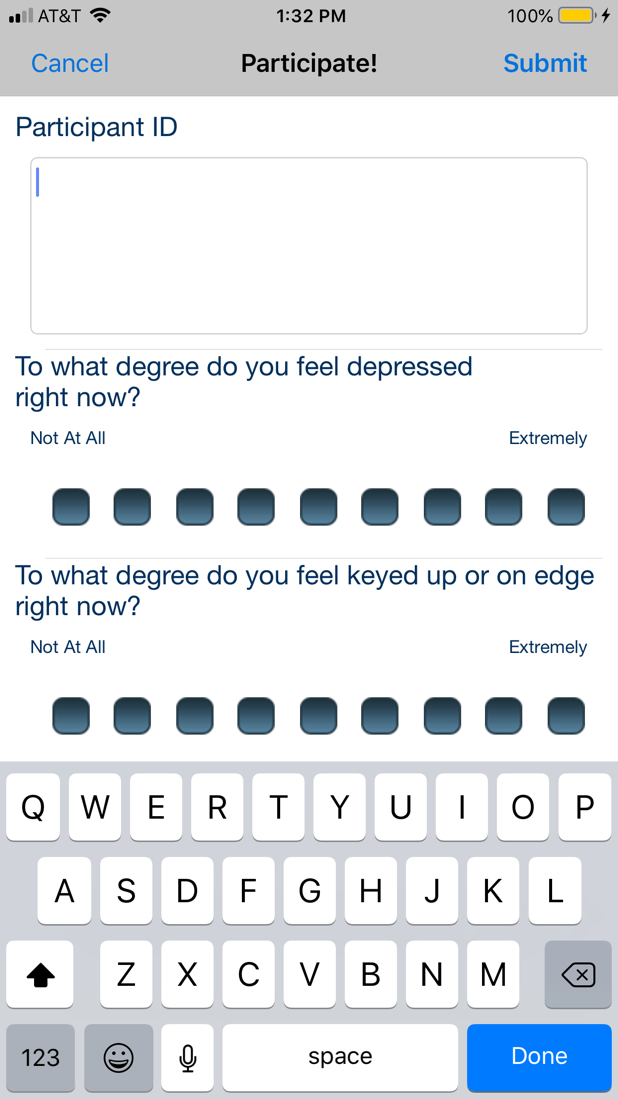

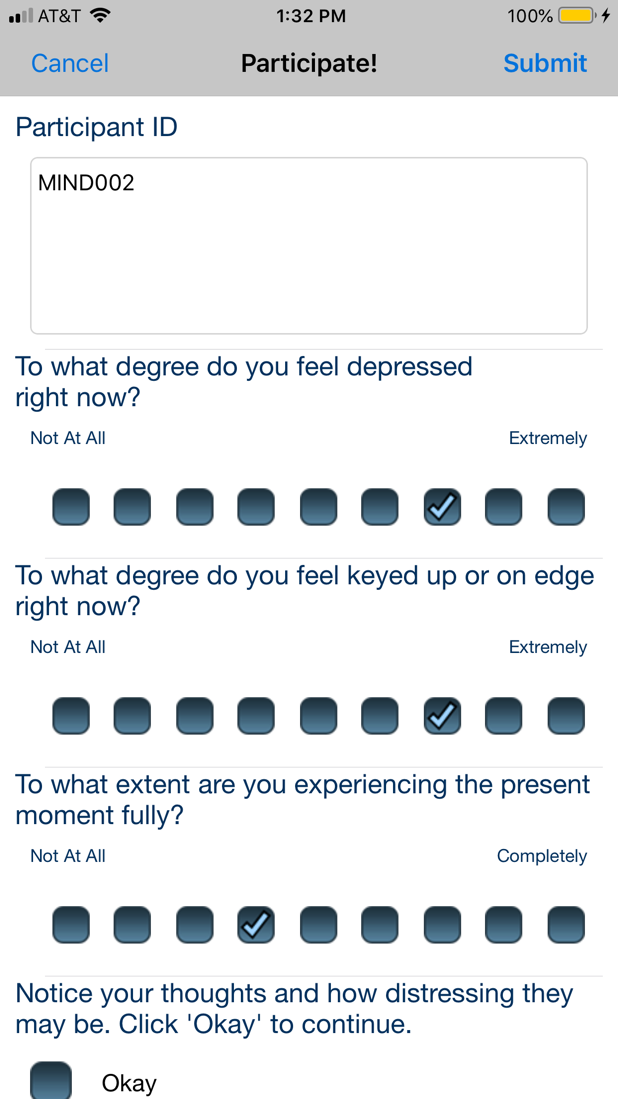


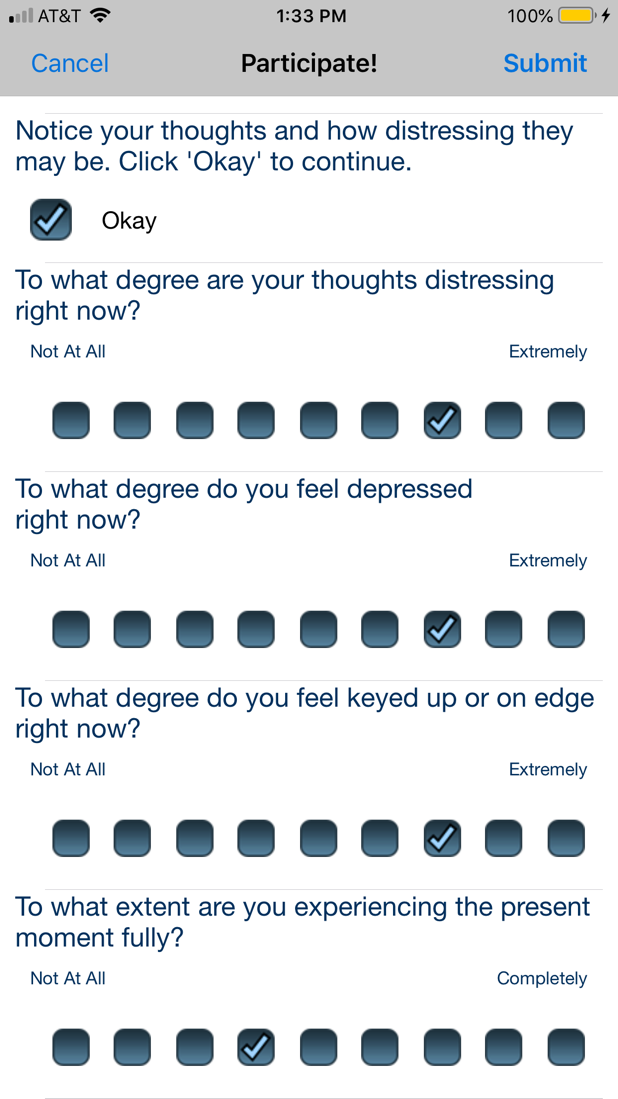

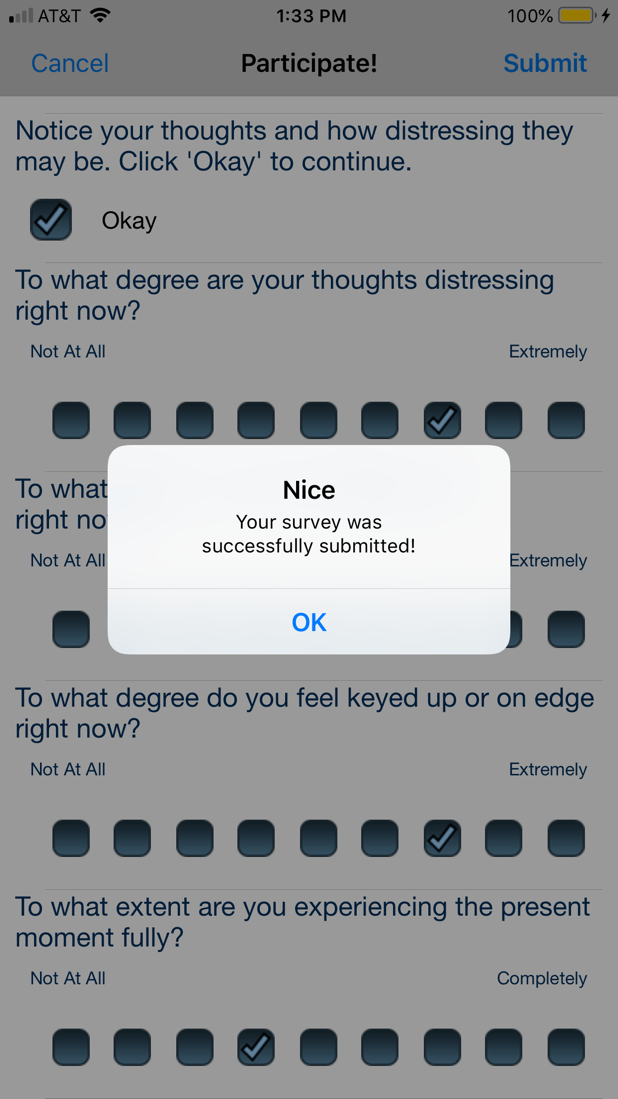


|  |  |  |  |  |  |  |
| --- | --- | --- | --- | --- | --- | --- |
|  | |  |  |  |  |  |
|  |  |  |  |  |  |  |
|  |  |  |  |  |  |  |
|  |  |  |  |  |  |  |
|  |  |  |  |  |  |  |
|  | |  |  |  |  |  |
|  |  |  |  |  |  |  |
|  |  |  |  |  |  |  |
|  |  |  |  |  |  |  |
|  |  |  |  |  |  |  |
|  | |  |  |  |  |  |
|  |  |  |  |  |  |  |
|  |  |  |  |  |  |  |
|  |  |  |  |  |  |  |
|  |  |  |  |  |  |  |
|  | |  |  |  |  |  |
|  |  |  |  |  |  |  |
|  |  |  |  |  |  |  |
|  |  |  |  |  |  |  |
|  |  |  |  |  |  |  |
|  | | |  |  |  |  |
|  |  |  |  |  |  |  |
|  |  |  |  |  |  |  |
|  |  |  |  |  |  |  |
|  |  |  |  |  |  |  |
|  | | |  |  |  |  |
|  |  |  |  |  |  |  |
|  |  |  |  |  |  |  |
|  |  |  |  |  |  |  |
|  |  |  |  |  |  |  |

|  |  |  |  |  |
| --- | --- | --- | --- | --- |
|  |  |  |  |  |
|  |  |  |  |  |
|  |  |  |  |  |
|  |  |  |  |  |
|  |  |  |  |  |
|  |  |  |  |  |
|  |  |  |  |  |
|  |  |  |  |  |
|  |  |  |  |  |
|  |  |  |  |  |
|  |  |  |  |  |
|  |  |  |  |  |
|  |  |  |  |  |
|  |  |  |  |  |
|  |  |  |  |  |
|  |  |  |  |  |
|  |  |  |  |  |
|  |  |  |  |  |
|  |  |  |  |  |
|  |  |  |  |  |

References

American Psychiatric Association. (2013). *Diagnostic and Statistical Manual of Mental Disorders* (5th ed.). APA Press. <https://doi.org/10.1176/appi.books.9780890425596>

Arend, M. G., & Schafer, T. (2019). Statistical power in two-level models: A tutorial based on Monte Carlo simulation. *Psychological Methods*, *24*(1), 1-19. <https://doi.org/10.1037/met0000195>

Brown, T. A., & Barlow, D. H. (2014). *Anxiety and related disorders interview schedule for DSM-5 (ADIS-5L): Client interview schedule*. Oxford University Press.

Devilly, G. J., & Borkovec, T. D. (2000). Psychometric properties of the credibility/expectancy questionnaire. *Journal of Behavior Therapy and Experimental Psychiatry*, *31*(2), 73-86. <https://doi.org/10.1016/S0005-7916(00)00012-4>

Enders, C. K., Du, H., & Keller, B. T. (2020). A model-based imputation procedure for multilevel regression models with random coefficients, interaction effects, and nonlinear terms. *Psychol Methods*, *25*(1), 88-112. <https://doi.org/10.1037/met0000228>

Gordon, D., & Heimberg, R. G. (2011). Reliability and validity of DSM-IV generalized anxiety disorder features. *Journal of Anxiety Disorders*, *25*(6), 813-821. <https://doi.org/10.1016/j.janxdis2011.04.001>

Graham, J. W. (2009). Missing data analysis: Making it work in the real world. *Annual Review of Psychology*, *60*(1), 549–576. <https://doi.org/10.1146/annurev.psych.58.110405.085530>

Homack, S., Lee, D., & Riccio, C. A. (2005). Test review: Delis-Kaplan Executive Function System. *Journal of Clinical and Experimental Neuropsychology*, *27*(5), 599-609. <https://doi.org/10.1080/13803390490918444>

Magnusson, K. (2018). powerlmm: Power analysis for longitudinal multilevel models. R package version 0.4.0. <https://CRAN.R-project.org/package=powerlmm>.

Martínez-Loredo, V., Fernández-Hermida, J. R., Carballo, J. L., & Fernández-Artamendi, S. (2017). Long-term reliability and stability of behavioral measures among adolescents: The Delay Discounting and Stroop tasks. *Journal of Adolescence*, *58*, 33-39. <https://doi.org/10.1016/j.adolescence.2017.05.003>

Moore, M. T., Anderson, N. L., Barnes, J. M., Haigh, E. A., & Fresco, D. M. (2013). Using the GAD-Q-IV to identify generalized anxiety disorder in psychiatric treatment seeking and primary care medical samples. *Journal of Anxiety Disorders*, *28*(1), 25-30. <https://doi.org/10.1016/j.janxdis.2013.10.009>

Moore, M. T., Anderson, N. L., Barnes, J. M., Haigh, E. A. P., & Fresco, D. M. (2014). Using the GAD-Q-IV to identify generalized anxiety disorder in psychiatric treatment seeking and primary care medical samples. *Journal of Anxiety Disorders*, *28*(1), 25-30. <https://doi.org/10.1016/j.janxdis.2013.10.009>

Newman, M. G., Zuellig, A. R., Kachin, K. E., Constantino, M. J., Przeworski, A., Erickson, T., & Cashman-McGrath, L. (2002). Preliminary reliability and validity of the Generalized Anxiety Disorder Questionnaire-IV: A revised self-report diagnostic measure of generalized anxiety disorder. *Behavior Therapy*, *33*(2), 215-233. <https://doi.org/10.1016/S0005-7894(02)80026-0>

Rutter, L. A., & Brown, T. A. (2015). Reliability and validity of the dimensional features of generalized anxiety disorder. *Journal of Anxiety Disorders*, *29*, 1-6. <https://doi.org/10.1016/j.janxdis.2014.10.003>

Shankman, S. A., Funkhouser, C. J., Klein, D. N., Davila, J., Lerner, D., & Hee, D. (2018). Reliability and validity of severity dimensions of psychopathology assessed using the Structured Clinical Interview for DSM-5 (SCID). *International Journal of Methods in Psychiatric Research*, *27*(1). <https://doi.org/10.1002/mpr.1590>

Spitzer, R. L., Williams, J. B. W., Kroenke, K., Linzer, M., deGruy, F. V., 3rd, Hahn, S. R., . . . Johnson, J. G. (1994). Utility of a new procedure for diagnosing mental disorders in primary care: The PRIME-MD 1000 study. *Journal of the American Medical Association*, *272*(22), 1749-1756. <https://doi.org/10.1001/jama.1994.03520220043029>

Wade, E. C., Cohen, R. T., Loftus, P., & Ruscio, A. M. (2022). A novel measure of real-time perseverative thought. *Clinical Psychological Science*, *10*(3), 534-552. <https://doi.org/10.1177/21677026211038017>
